# A Case of an Anomalous Right Coronary Artery Arising from the Left Coronary Cusp

**DOI:** 10.7759/cureus.4659

**Published:** 2019-05-14

**Authors:** Muhammad Shabbir Rawala, Ahmer A Longi, Arfeen A Khan, Syed Imran Rizvi, Syed B Rizvi

**Affiliations:** 1 Internal Medicine, Charleston Area Medical Center, Charleston, USA; 2 Internal Medicine, Aga Khan University Hospital, Karachi, PAK; 3 Internal Medicine, Dr. Ziauddin University and Hospital, Karachi, PAK; 4 Cardiac Surgery, Bristol Royal Infirmary, Bristol, GBR; 5 Cardiology, Rapides Regional Medical Center, Alexandria, USA

**Keywords:** congenital anomaly, chest pain, coronary artery

## Abstract

An anomalous origin of the right coronary artery is usually asymptomatic. It is mostly found incidentally on an invasive diagnostic angiogram. It does lead to an increased risk of sudden cardiac death, especially in younger patients. We present a case of a 41-year-old who had presented to the hospital with complaints of chest pain. The patient was evaluated by cardiology who performed an angiography that identified an anomalous origin of the right coronary artery arising from the left coronary cusp but no evidence of coronary artery disease. Once identified, these anomalous vessels should be corrected surgically, as these conditions increase the risk of sudden cardiac death arrhythmia and ischemic events.

## Introduction

Approximately 1% of patients who undergo cardiac catheterization are reported to have coronary artery anomalies [1]. The incidence of an anomalous right coronary artery originating from the left coronary sinus on coronary angiography is 0.019% to 0.49% [1]. Often, patients present with sudden death or myocardial ischemia [2]. Most patients remain asymptomatic and have no ischemic symptoms or findings on resting or stress electrocardiography (ECG); therefore, they are diagnosed only on coronary imaging [3]. We report a case of a 41-year-old female admitted with complaints of chest pain, who underwent catheterization that detected an anomalous right coronary artery arising from the left coronary cusp.

## Case presentation

**Figure 1:**
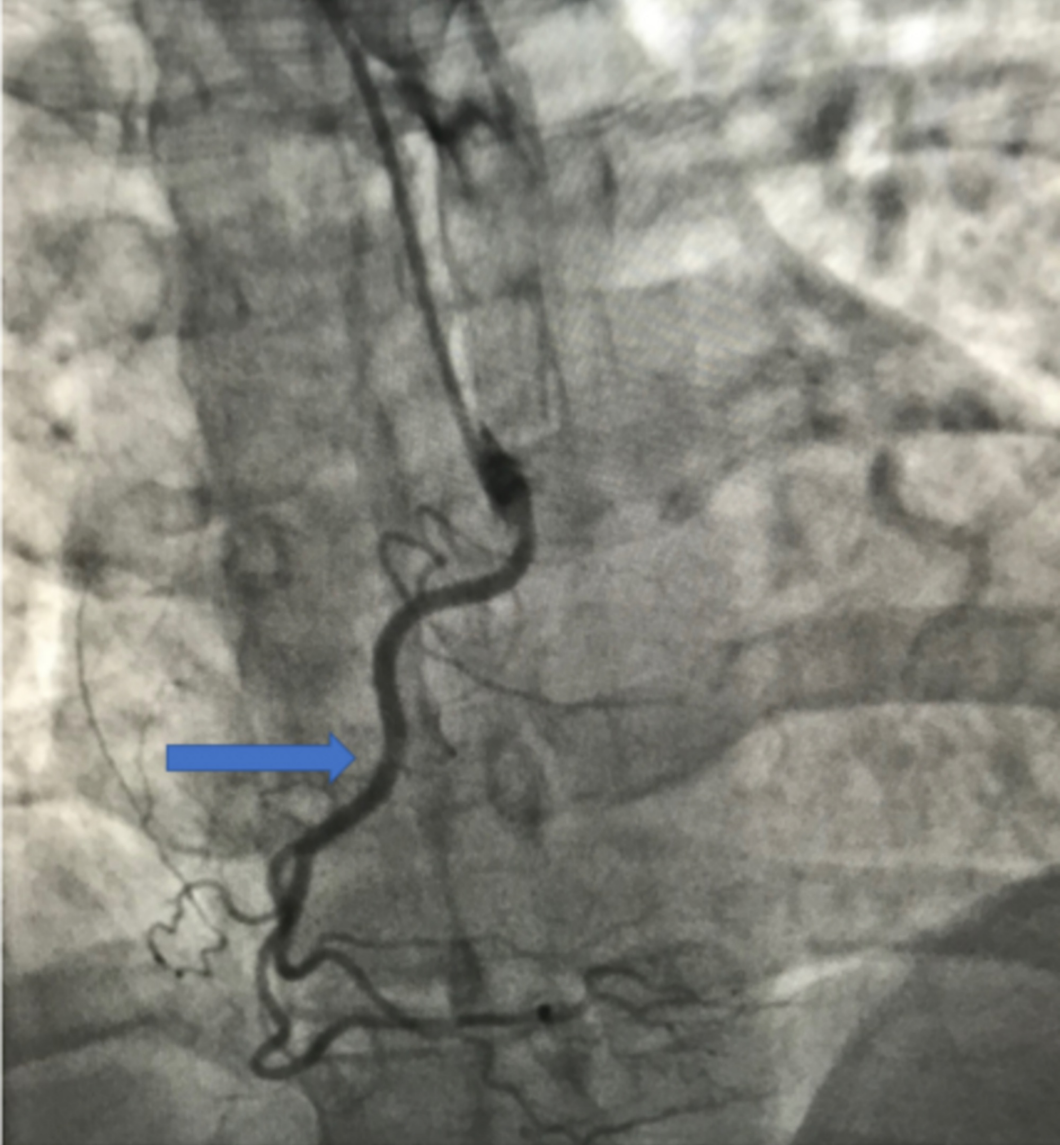
Invasive angiogram demonstrating anomalous right coronary artery (arrow)

**Figure 2:**
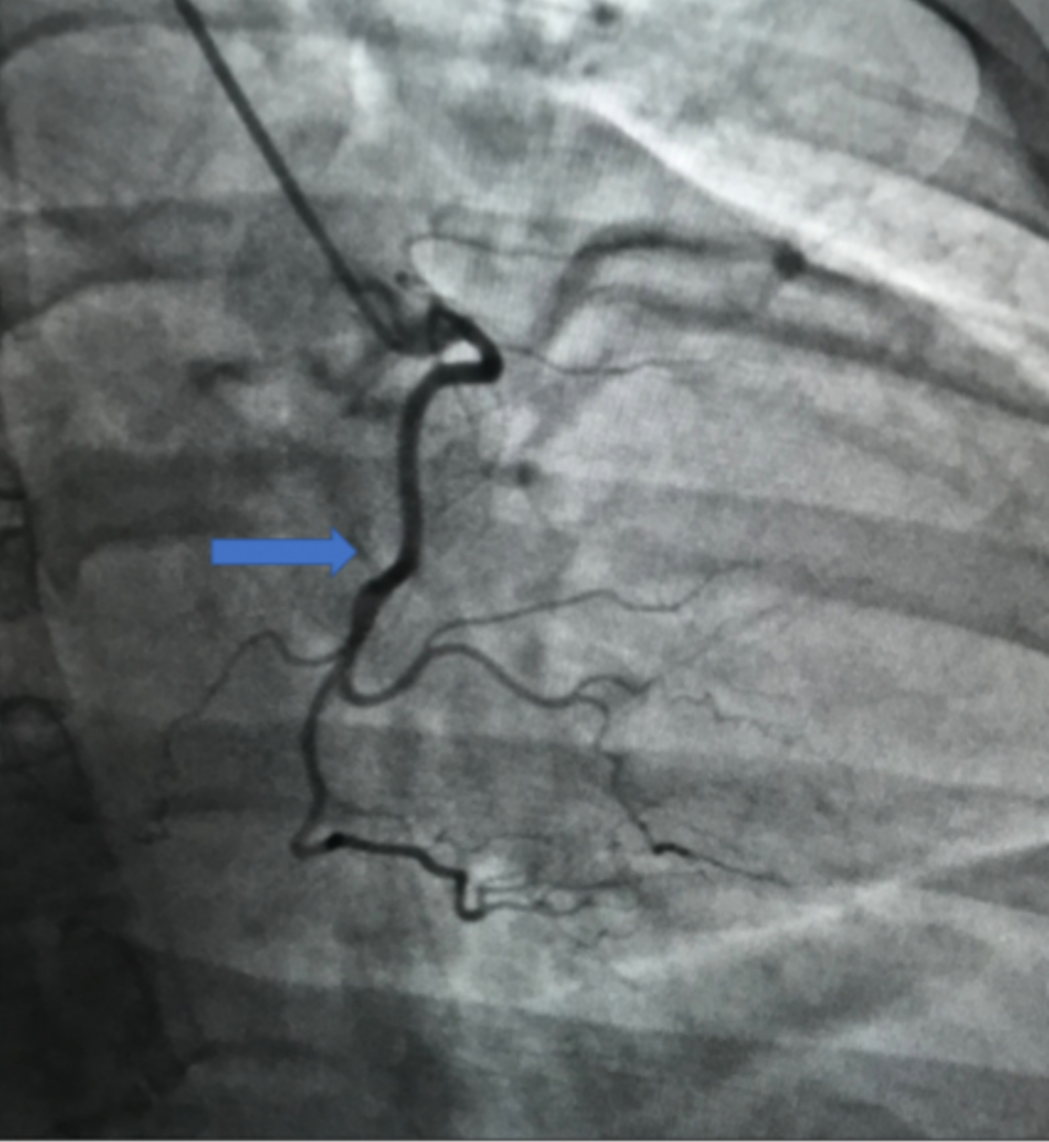
Invasive angiogram demonstrating anomalous right coronary artery (arrow)

A 41-year-old African American female with a history of hypertension presented to the emergency department (ED) with complaints of chest pain. The patient had experienced intermittent chest pain throughout the night but presented to the ED in the morning after having chest pain along with shortness of breath. On examination, the patient’s vitals were stable, and the remaining systemic examination was unremarkable. Cardiology service evaluated the patient; an echocardiogram revealed the left ventricular ejection fraction to be 65% with no valvular abnormalities. The patient’s troponins were negative; however, due to the patient’s family history of premature coronary artery disease and the risk factor of hypertension, cardiology decided to evaluate the patient further using invasive angiography (IA). IA identified an anomalous right coronary artery arising from the left coronary cusp (Figures 1-2) and having an anterior inter-arterial course with no evidence of coronary artery disease. The patient was transferred to a tertiary care center to be evaluated by cardiothoracic surgery for correction of the anomaly.

## Discussion

An anomalous origin of the right coronary artery (RCA) is a rare congenital anomaly that was first described in 1948 by White and Edwards [4]. After carrying out angiography in 126,595 patients, Yamanaka and Hobbs reported the incidence of anomalous origin of the right and left coronary arteries as 136 (0.107%) and 22 (0.017%), respectively [5]. The prevalence of an anomalous origin of RCA (ARCA) arising from the left coronary cusp with an inter-arterial course varies between 0.026% and 0.25% [6-7]. An ARCA is more common than the anomalous origin of the left coronary artery (ALCA), but the latter is shown to be responsible for up to 85% of sudden cardiac deaths (SCD) related to the anomalous origins of arteries [8].

Most coronary anomalies are detected incidentally on diagnostic angiography and are clinically insignificant; some, however, have been associated with an increased risk of SCD. It is second only to hypertrophic cardiomyopathy as a leading cause of SCD in young athletes. Mechanical compression of the RCA by the great vessels, which dilate and compress the RCA during periods of increased stroke volume, is the usual explanation for coronary ischemia [9]. Patients usually have no ischemic changes on an electrocardiogram (ECG) at rest; however, multi-detector computed tomography (MDCT) is being used to evaluate the course of anomalous vessels outlying their relative positions to the aorta and the pulmonary artery. It also gives additional information regarding any stenotic lesion in these anomalous vessels [3]. Echocardiography (Echo), magnetic resonance angiography (MRA), MCDT, and cardiac catheterization are all complementary diagnostic tools for evaluating anomalous coronary arteries [10].

While it is agreed that surgical correction is the standard of care for ALCA, when found, the management for right ARCA is more complicated. Treatment options for these patients include observation with medical therapy, percutaneous intervention (stenting), or surgery [11]. Early diagnosis of coronary artery anomalies based on the patients’ symptoms can help decrease the incidence of sudden cardiac deaths, fatal arrhythmias, and ischemic events in the high-risk population.

## Conclusions

Our patient had an anomalous right coronary artery arising from the left coronary cusp, with no evidence of coronary artery disease. It is judicious to understand the anatomy of anomalous vessels beforehand if a cardiac intervention is to be planned, as this may help direct the approach of intervention. Anomalous vessels may lead to sudden cardiac death, arrhythmias, or ischemic events and, therefore, should be corrected when symptomatic.
